# Safety perspectives on presently considered drugs for the treatment of COVID‐19

**DOI:** 10.1111/bph.15204

**Published:** 2020-08-13

**Authors:** Sophie L. Penman, Robyn T. Kiy, Rebecca L. Jensen, Christopher Beoku‐Betts, Ana Alfirevic, David Back, Saye H. Khoo, Andrew Owen, Munir Pirmohamed, B. Kevin Park, Xiaoli Meng, Christopher E. Goldring, Amy E. Chadwick

**Affiliations:** ^1^ MRC Centre for Drug Safety Science, Department of Pharmacology and Therapeutics University of Liverpool Liverpool UK; ^2^ School of Medicine University of Liverpool Liverpool UK

**Keywords:** COVID‐19, drug repurposing, drug safety, toxicology

## Abstract

Intense efforts are underway to evaluate potential therapeutic agents for the treatment of COVID‐19. In order to respond quickly to the crisis, the repurposing of existing drugs is the primary pharmacological strategy. Despite the urgent clinical need for these therapies, it is imperative to consider potential safety issues. This is important due to the harm–benefit ratios that may be encountered when treating COVID‐19, which can depend on the stage of the disease, when therapy is administered and underlying clinical factors in individual patients. Treatments are currently being trialled for a range of scenarios from prophylaxis (where benefit must greatly exceed risk) to severe life‐threatening disease (where a degree of potential risk may be tolerated if it is exceeded by the potential benefit). In this perspective, we have reviewed some of the most widely researched repurposed agents in order to identify potential safety considerations using existing information in the context of COVID‐19.

**Abbreviations**
AAK1: AP2‐associated protein kinase 1
ADE: antibody‐dependent enhancement
ADR: adverse drug reaction
AEs: adverse events
ALT: alanine aminotransferase
ANC: absolute neutrophil count
ARB: angiotensin receptor blocker
AST: aspartate transaminase
*C*_max_
: maximum serum concentration
COPD: chronic obstructive pulmonary disease
CYP: cytochrome P450
DDI: drug–drug interaction
DILI: drug‐induced liver injury
DMARD: disease‐modifying anti‐rheumatic drug
DVT: deep vein thrombosis
EAMS: Early Access to Medicines Scheme
ExoN: exoribonuclease
favipiravir‐RTP: favipiravir‐ribofuranosyl‐5′‐triphosphate
HCoV: human coronavirus
HIV: human immunodeficiency virus
ICU: intensive care unit
IRF: IFN regulatory factor
MCD: multicentric Castleman disease
MERS‐CoV: Middle East respiratory syndrome coronavirus
MHRA: Medicines and Healthcare products Regulatory Agency
M^pro^
: main protease
MS: multiple sclerosis
NSAIDs: non‐steroidal anti‐inflammatory drugs
nsp14: non‐structural protein 14
P‐gp: P‐glycoprotein
PK: pharmacokinetic
RdRp: RNA‐dependent RNA polymerase
SARS‐CoV‐2: severe acute respiratory syndrome coronavirus 2
SBECD: sulfobutylether β‐cyclodextrin sodium
sHLH: secondary haemophagocytic lymphohistiocytosis
TMA: thrombotic microangiopathy
ULN: upper limit of normal
WHO: World Health Organization

## 1 INTRODUCTION

**FIGURE 1:**
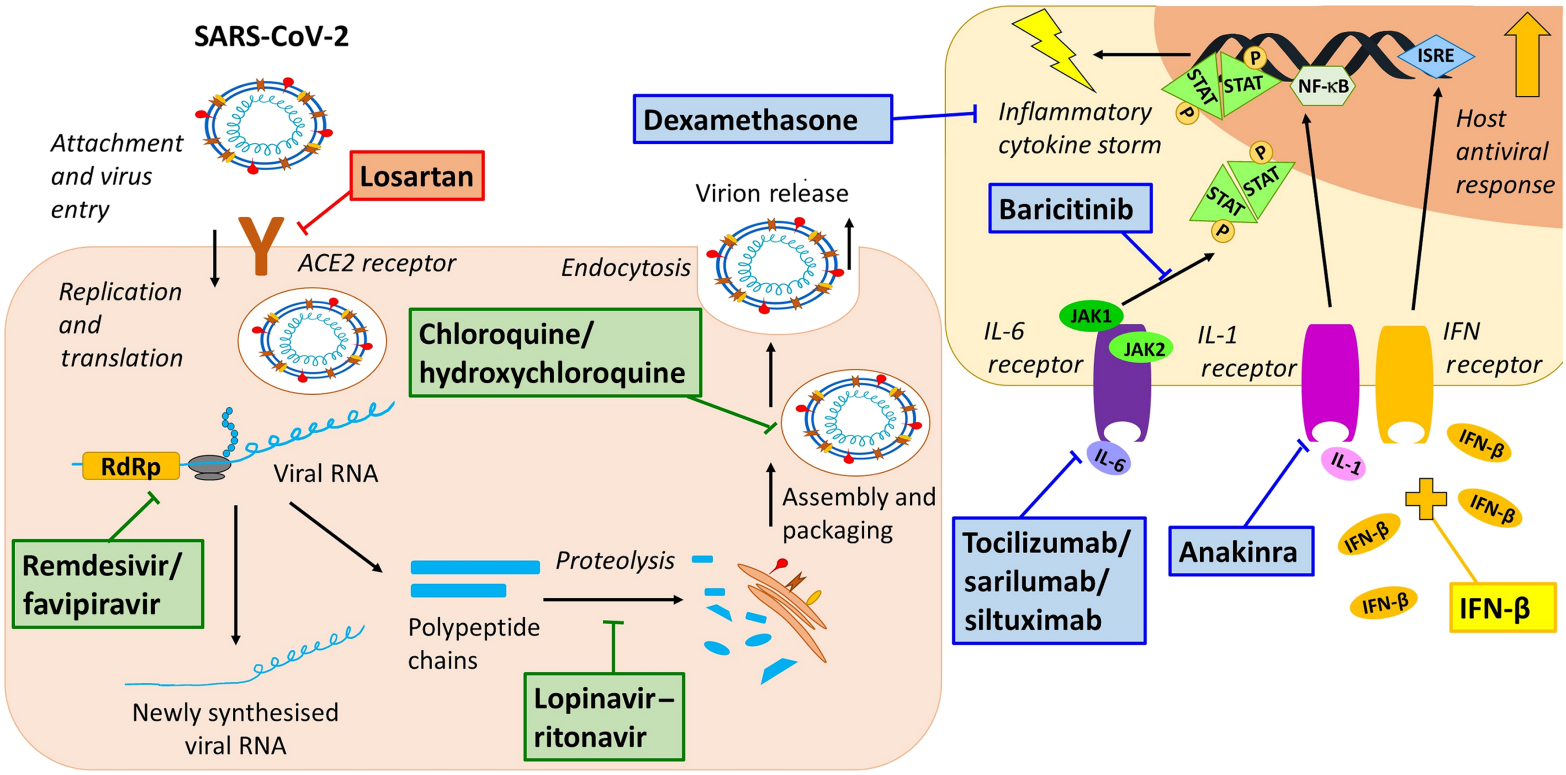
Overview of the mechanisms of action of the repurposed drugs undergoing clinical trials for the treatment of COVID‐19 that are reviewed here. Compounds in red represent those that are viral entry inhibitors, compounds in green represent disruptors of cellular viral processing, compounds in blue are modulators of the hyperinflammatory phase of infection, and compounds in yellow stimulate host immunomodulatory and antiviral activity. ISRE, IFN‐stimulated response element; P, phosphate; RdRp, RNA‐dependent RNA polymerase; SARS‐CoV‐2, severe acute respiratory syndrome coronavirus 2

Severe acute respiratory syndrome coronavirus 2 (SARS‐CoV‐2) is a highly pathogenic betacoronavirus that emerged in Wuhan, Hubei Province, in late December 2019 (Yang, Liu, et al., 2020; Yang, Yu, et al., 2020). SARS‐CoV‐2 is the seventh human coronavirus (HCoV) to be identified and is the cause of the disease known as COVID‐19, which was declared by the World Health Organization (WHO) as a “Public Health Emergency of International Concern” on January 30, 2020 (Liu, Hu, et al., 2020). The symptoms of COVID‐19 are non‐specific and cover a broad clinical spectrum, meaning that clinical diagnosis without a test is challenging. Patients commonly present with fever, cough, and anosmia, although many patients are asymptomatic. The virus can be transmitted by asymptomatic patients alongside those in the symptomatic and pre‐symptomatic phase of the disease (Wu, Wu, Liu, & Yang, 2020; Yang, Yu, et al., 2020). As of July 8, 2020, COVID‐19 was responsible for 539,057 deaths worldwide (John Hopkins University and Medicine, 2020). As the number of new cases continues to increase rapidly, many clinical and preclinical studies have been initiated to identify viable treatment options for COVID‐19 patients. Many of these potential therapeutic strategies are based upon the repurposing of approved drugs or the evaluation of those currently in the clinical stages of drug development (Figure 1). For this reason, much information already exists on the pharmacology and toxicology of each prospective therapy. In order to examine their potential for efficacy and safety against COVID‐19, it is essential to consider all of the available information in this extremely fast‐moving and critical research field. Therefore, it is important to assess drug‐specific safety parameters in the context of the pathogenesis of the virus and its clinical features in order to begin to evaluate the disease‐specific harm–benefit ratio.

Therapeutic strategies for the treatment of COVID‐19 span a range of phases including prophylactic administration, through to early infection, through to the more severe disease stage characterised by pulmonary involvement requiring mechanical ventilation and hyperinflammation in some instances (Siddiqi & Mehra, 2020). At the time of writing, over 2,400 clinical trials have been initiated across the globe, with the majority of these investigating the use of drug(s) already approved for treatment of another disease (ClinicalTrials.gov, 2020b). In addition to administration as a monotherapy, combination therapy has also been utilised. In addition to repurposed compounds, several candidates in preclinical or clinical development stages are also under investigation, as well as widely available borderline substances such as ascorbic acid (ClinicalTrials.gov, 2020d). However, it is important to note that the number of clinical trials initiated should not be taken as an indicator of the ultimate potential for success.

Our perspective will focus upon *selected* repurposed therapies, which are used in different phases of the disease (Figure 1) and can broadly be categorised by their mechanism of action as (a) viral entry inhibitors, (b) disruption of cellular viral processing, (c) modulators of the inflammatory phase, and (d) compounds that stimulate the host's antiviral response. Selections were based upon agents undergoing the largest clinical trials at the time of writing, July 8, 2020.

## 2 INHIBITORS OF ENTRY INTO EPITHELIAL CELLS

It has been widely reported that ACE2 is likely to be the predominant cell surface receptor by which SARS‐CoV‐2 is able to enter host cells (Hoffmann et al., 2020). ACE2 is widely distributed in the body, but is particularly highly expressed on lung alveolar cells, which could explain the increased lung damage seen in some COVID‐19 patients (Favalli et al., 2020; Zhao et al., 2020).

### 2.1 Angiotensin receptor blockers

Losartan is an antagonist of AT_1_ receptors, one of the angiotensin receptor blockers (ARBs) widely used as anti‐hypertensive drugs. In the present context, losartan is hypothesised to block the attachment of SARS‐CoV‐2 to cells expressing ACE2, thereby preventing viral entry into host cells (Gurwitz, 2020). Losartan is a well‐tolerated compound used in the treatment of hypertension, heart failure and diabetes (Goa & Wagstaff, 1996; Ripley & Hirsch, 2010). For these indications, losartan treatment is lifelong and is typically administered at a dose of 25–50 mg once a day, which can be increased to 100 mg if tolerated and deemed necessary (Dohme, 2003). Clinical trials for losartan use in COVID‐19 are currently recruiting patients. While exact dosing regimens vary between the trials, they are based upon the standard dose and will see patients given 25–50 mg daily losartan treatment for 7–14 days (Clinical Trial Identifiers NCT04335123, NCT04312009, and NCT04311177).

When used for its intended therapeutic conditions, losartan is considered to be a relatively safe compound. The most common adverse events (AEs) reported during clinical trials were headache, dizziness, fatigue, and hypotension (Goa & Wagstaff, 1996; Usai et al., 2020; Weber, 1997). Laboratory findings during clinical trials reported mild elevations in alanine aminotransferase (ALT), but these were self‐limiting and did not result in acute liver failure (Goa & Wagstaff, 1996). More recently, cases of losartan‐induced hepatotoxicity have been reported, albeit rarely (LiverTox, 2017a). Losartan is associated with serious cases of fetal toxicity and so cannot be administered to women in the second and third trimesters of pregnancies (Nayar, Singhal, Aggarwal, & Malhotra, 2003).

There is controversy regarding the use of losartan in COVID‐19 trials as the expression of ACE2 can be significantly increased following ARB treatment. However, contradictory results for the potential of ARB‐induced ACE2 up‐regulation have also been reported (Ferrario et al., 2005; Klimas et al., 2015; Tignanelli et al., 2020). Given that ACE2 is already highly expressed within the lungs, a further increase in ACE2 expression may have the potential to increase viral entry into the lungs, thus exacerbating the potential for pulmonary involvement (Fang, Karakiulakis, & Roth, 2020). Evidence of increased myocardial injury and chronic damage to the cardiovascular system has been reported in patients infected with SARS‐CoV‐2 (Zheng, Ma, Zhang, & Xie, 2020). ACE2 is also expressed within the cardiovascular system, and it has been suggested that this may underlie cardiac effects via enhanced viral entry into the myocardium (Zheng et al., 2020). It is important to note that human data thus far do not support the hypothesis that ARBs (and ACE inhibitors) increase the risk of severe COVID‐19 disease because epidemiological studies have largely shown either a protective effect or no adverse effects on mortality in COVID‐19 (Tignanelli et al., 2020). Importantly, the latest advice from the Medicines and Healthcare products Regulatory Agency (MHRA, 2020a) recommends that those taking ARBs and ACE inhibitors for high BP should continue doing so. Data from clinical trials for losartan in COVID‐19 will provide additional safety data and much needed clarification on the role of ARBs and ACE2 in COVID‐19.

Losartan has an overall favourable drug–drug interaction (DDI) profile. However, concerns over the use of losartan and non‐steroidal anti‐inflammatory drugs (NSAIDs) during COVID‐19 treatment have been raised (Sica, Gehr, & Ghosh, 2005). Evidence initially suggested that there was a theoretical potential for NSAIDs to worsen COVID‐19 symptoms. However, the Commission on Human Medicines deemed that there was insufficient evidence to confirm this association and that NSAIDs were safe to use in individuals with symptoms of COVID‐19 (Commission on Human Medicines, 2020; Torjesen, 2020). However, NSAIDs have the potential to attenuate the effects of losartan to reduce hypertension, thereby leading to an increase in BP, which would be an undesirable side effect in COVID‐19 patients with hypertension, lung inflammation, and cardiac damage (Pavlicević, Kuzmanić, Rumboldt, & Rumboldt, 2008). In such patients, BP should be closely monitored.

## 3 DISRUPTORS OF CELLULAR VIRAL PROCESSING

### 3.1 Lopinavir–ritonavir

Lopinavir–ritonavir is a fixed dose combination of anti‐viral drugs branded as Kaletra® and developed for the treatment of human immunodeficiency virus (HIV) disease. Lopinavir is a protease inhibitor that is co‐administered with ritonavir to improve the pharmacokinetic (PK) properties of the drug (Chu et al., 2004). Lopinavir–ritonavir is a potential treatment for COVID‐19 as it targets and deactivates the SARS‐CoV‐2 main protease (M^pro^), which is involved in polyprotein processing and virus maturation (Dayer, Taleb‐Gassabi, & Dayer, 2017). A standard dose of lopinavir–ritonavir is 400/100 mg twice a day for HIV‐1 treatment, and this has also been used for SARS‐CoV‐2 treatment (Cao et al., 2020).

The most frequently reported AEs for lopinavir–ritonavir treatment are gastrointestinal disturbances including diarrhoea, nausea, and vomiting (Chandwani & Shuter, 2008). Dose‐related diarrhoea has been reported in up to 25% of patients and is thought to occur through a number of mechanisms including decreased proliferation of intestinal epithelial cells, disruption of intestinal barrier function, inducing endoplasmic reticulum stress, and activating the unfolded protein response (Wu, Li, Peng, & Zhou, 2014). Diarrhoea is also a symptom in some COVID‐19 patients, and so lopinavir–ritonavir has the potential to exacerbate this. Pancreatitis has been reported in a small number of patients following lopinavir–ritonavir treatment, although this was more frequent in those with a pre‐existing history of pancreatitis (Chandwani & Shuter, 2008; Oldfield & Plosker, 2006). Additionally, patients with underlying liver diseases should have regular monitoring of hepatic function (Palacios et al., 2006). Caution should be exerted for those patients taking concomitant medication as lopinavir–ritonavir inhibits P‐glycoprotein (P‐gp) and cytochrome P450 (CYP)‐3A4, which therefore may alter the PK of other compounds (Zhang, Zhang, & Huang, 2009). A COVID‐19 drug interaction website has been developed by the Liverpool Drug Interaction Group, which details the drug–drug interactions (DDIs) found with lopinavir–ritonavir and a number of drugs, which in some cases can lead to potentially serious and/or life‐threatening reactions (AbbVie Inc., 2016; Liverpool Drug Interaction Group, 2020).

Since the SARS‐CoV‐2 outbreak, 79 clinical trials have been registered (up to July 8, 2020) to test lopinavir–ritonavir as a potential treatment for SARS‐CoV‐2 with variable outcomes in terms of efficacy. In one trial of 199 patients with confirmed SARS‐CoV‐2, 13 patients on the lopinavir–ritonavir arm were withdrawn due to AEs (Cao et al., 2020). In a different trial, patients who were administered with lopinavir–ritonavir (200/50 mg) also experienced gastrointestinal AEs (Li et al., 2020).

### 3.2 Chloroquine and hydroxychloroquine

Chloroquine and its derivative, hydroxychloroquine, are widely used as inexpensive and safe antimalarial drugs. In particular, the established good tolerability of chloroquine/hydroxychloroquine has made them safe to use even in pregnancy (Villegas et al., 2007). In addition to antimalarial activity, both drugs have immunomodulating effects and are used for the treatment of autoimmune diseases including systemic and discoid lupus erythematosus, psoriatic arthritis and rheumatoid arthritis. Chloroquine/hydroxychloroquine concentrates extensively in acidic vesicles including the endosomes, Golgi vesicles, and the lysosomes (Ohkuma & Poole, 1981). This leads to lysosomal membrane permeabilisation or dysfunction of several enzymes including acid hydrolases and palmitoyl protein thioesterase 1 (Rebecca et al., 2019; Savarino, Boelaert, Cassone, Majori, & Cauda, 2003; Schrezenmeier & Dorner, 2020). Although the precise mechanisms of the antiviral effects are not fully understood, it has been proposed that chloroquine/hydroxychloroquine can prevent virus infection (pre‐infection) by interfering with the glycosylation of cellular receptors and impair viral replication by increasing endosomal pH (post‐infection) (Savarino et al., 2003; Savarino et al., 2004; Vincent et al., 2005).

Owing to their efficacy against viruses (mostly demonstrated in vitro) including influenza, HIV, coronavirus OC43, and SARS‐CoV, a large number of clinical trials (>230) have been registered worldwide using chloroquine/hydroxychloroquine alone, or in combination with other drugs (e.g., azithromycin) for the treatment of COVID‐19. Despite promising in vitro antiviral results for hydroxychloroquine/chloroquine, there is no convincing evidence of efficacy in vivo at present (Gao, Tian, & Yang, 2020; Gautret et al., 2020a; Gautret et al., 2020b; Liu et al., 2020; Magagnoli et al., 2020; Mathian et al., 2020; Million et al., 2020; Tang et al., 2020; Yao et al., 2020). A post‐exposure prophylaxis randomised controlled trial of 821 participants failed to show any benefit of hydroxychloroquine (*n* = 414) compared with placebo (*n* = 407) (Boulware et al., 2020). At the time of writing, the RECOVERY trial (Clinical Trial Identifier NCT04381936), which is the largest randomised control trial so far conducted for the treatment of COVID, has stopped recruiting to the hydroxychloroquine arm (1,542 patients compared with 3,132 on standard care) because of no beneficial effect in terms of either mortality or hospital stay (Horby & Landray, 2020). There are still many other trials ongoing testing the efficacy of hydroxychloroquine for either prophylaxis or treatment.

Both chloroquine and hydroxychloroquine have been in clinical use for many years for rheumatoid diseases, and thus, their safety profile is well established. Dose‐dependent retinal toxicity has long been recognised as the major AE with long‐term use of chloroquine/hydroxychloroquine (Marmor et al., 2011). Besides retinal toxicity, gastrointestinal, liver, and renal toxicity have also been reported (Giner Galvan, Oltra, Rueda, Esteban, & Redon, 2007; Michaelides, Stover, Francis, & Weleber, 2011; Mittal, Zhang, Feng, & Werth, 2018). As both drugs are mainly metabolised in the liver and excreted by renal clearance, their use in patients with liver or renal impairment may worsen the function of these organs. For chloroquine treatment, prescribing information recommends the full dose at all degrees of renal impairment but suggests that monitoring of renal function may be useful (Sanofi‐Aventis, 2017a). For hydroxychloroquine, reductions in dosage are advised for patients with impaired renal function, as well as those taking concomitant medications with known risks of kidney damage (Concordia Pharmaceuticals Inc., 2017).

A serious AE associated with chloroquine/hydroxychloroquine is cardiotoxicity, which can take many forms including cardiomyopathy in rare instances. Prolonged treatment or high dosage of chloroquine/hydroxychloroquine has been shown to increase the risk of QT interval prolongation, polymorphic ventricular tachycardia, and sudden cardiac death (Chatre, Roubille, Vernhet, Jorgensen, & Pers, 2018). A large epidemiological analysis in patients with rheumatoid arthritis has recently shown that 30‐day cardiovascular mortality was increased by more than twofold when hydroxychloroquine was combined with azithromycin. The lethal ventricular arrhythmias are primarily due to inhibition of a potassium channel (the inward rectifier K_ir_2.1 channel) and may occur at low micromolar concentrations (IC_50_ = 8.7 μM) (Rodriguez‐Menchaca et al., 2008). While therapeutic doses of chloroquine typically result in plasma concentrations of 2–5 μM, much higher concentrations in the heart are expected based on a 400‐fold increase observed in rat PK studies (McChesney, Banks, & Fabian, 1967; Walker, Dawodu, Adeyokunnu, Salako, & Alvan, 1983). Both drugs act on various potassium channels including the inward rectifier currents (carried by K_ir_2.1 and K_ir_6.2 channels) and rapid delayed rectifier currents (carried by K_v_11.1/hERG channels) (Ponce‐Balbuena et al., 2012; Rodriguez‐Menchaca et al., 2008; Sánchez‐Chapula, Navarro‐Polanco, Culberson, Chen, & Sanguinetti, 2002). The binding of chloroquine to the inward rectifier K_ir_2.1 channel can be stabilised by negatively charged and aromatic amino acids (Rodriguez‐Menchaca et al., 2008). To a lesser extent, chloroquine also blocks the rapid delayed rectifier current, I_Kr_, possibly through cation–π and π‐stacking interactions with Tyr652 and Phe656 in the S6 domain of hERG channels (Sánchez‐Chapula et al., 2002). The effect of inhibition of these potassium channels on the heart rate appears to be complex. However, blocking the hERG channel has proven to be the most common mechanisms by which drugs cause QT interval prolongation (Traebert & Dumotier, 2005). The binding of chloroquine/hydroxychloroquine to proteins is also stereoselective, but whether one of the chloroquine/hydroxychloroquine enantiomers has a stronger interaction with the K_ir_2.1 channel is not known. Caution is needed when hydroxychloroquine is used in combination with other drugs (including azithromycin), which increase the QT interval because of a pharmacodynamic synergistic interaction.

Given the co‐morbidities in many patients with COVID‐19, especially those with underlying cardiovascular disease, and the fact that COVID‐19 itself is associated with cardiac manifestations, this may increase the risk of cardiotoxicity associated with the use of chloroquine/hydroxychloroquine. Indeed, excessive QTc prolongation was observed in 36% of patients reported by Bessiere at al., and greater QTc prolongation was also seen in patients taking the combination of hydroxychloroquine and azithromycin than those taking hydroxychloroquine alone, highlighting the importance of pharmacodynamic interactions (Bessiere et al., 2020; Mercuro et al., 2020). Furthermore, a Phase IIb trial in Brazil showed that a higher dose of chloroquine (600 mg twice daily) in patients hospitalised with COVID‐19 had a higher fatality rate (30%) compared with 15% in the lower dose (450 mg twice daily) group (Borba et al., 2020). QTc interval prolongation >500 ms was observed in 19% of the high‐dose group compared with 11% of the low‐dose group. The US prophylaxis randomised control trial however did not show any increase in cardiovascular AEs (Boulware et al., 2020). We await the publication of the RECOVERY trial to determine whether there was an excess of cardiovascular events. However, it is important to note that despite the size of the RECOVERY trial (*n* = 1,542 patients), it may still be underpowered to identify an excess number of cardiovascular events when compared with standard of care.

### 3.3 Remdesivir

Remdesivir is an investigational compound that was developed for the treatment of Ebola (Mullard, 2018; Tchesnokov, Feng, Porter, & Gotte, 2019). Remdesivir is a monophosphoramidate prodrug and acts as a broad‐spectrum antiviral that can be incorporated into viral RNA (Agostini et al., 2018; Sheahan et al., 2020; Warren et al., 2016). Many antivirals are proving to be ineffective against COVID‐19 due to the presence of a proofreading exoribonuclease (ExoN) specific to coronaviruses, encoded in non‐structural protein 14 (nsp14) (Agostini et al., 2018). Remdesivir is able to evade this viral proofreading, allowing its incorporation into viral RNA and resulting in inhibition of RNA‐dependent RNA polymerases (RdRps), thereby preventing subsequent viral replication (Warren et al., 2016). Furthermore, Arshad et al. (2020) suggest that the maximum serum concentration (*C*_max_) of remdesivir is sufficient to inhibit 90% of SARS‐CoV‐2 replication, a parameter that is suspected to be of vital importance in the treatment of COVID‐19.

Remdesivir is administered intravenously, with single doses ranging between 3 and 225 mg being well tolerated in Ebola patients (*n* = 8) (ClinicalTrials.gov, 2019). Similar observations were made in the blinded, placebo‐controlled multiple‐dose studies, during which Ebola patients (*n* = 8) received an intravenous infusion of 150‐mg remdesivir daily for either 7 or 14 days, and only Grade 1 and 2 adverse reactions were reported (ClinicalTrials.gov, 2019). The proposed dosing regimen for COVID‐19 patients receiving remdesivir via the UK Early Access to Medicines Scheme (EAMS) is similar to that was evaluated for Ebola treatment: a loading dose of 200 mg on Day 1, followed by 100 mg daily for 5–10 days depending on symptom severity (MHRA, 2020b). As such, it is likely that many of the AEs observed in the Ebola study will translate to COVID‐19 patients treated with remdesivir.

Mild‐to‐moderate elevation of ALT and aspartate transaminase (AST) were observed in several Ebola patients during the multiple‐dose study, reflecting observations made in human hepatocytes in vitro (ClinicalTrials.gov, 2019; WHO, 2018). This is likely to be due to the high cell permeability of hepatocytes, in combination with the effective intracellular metabolism of remdesivir to its active form within the liver (WHO, 2018). Emerging data have suggested that SARS‐CoV‐2 may target ACE2 on hepatocytes leading to liver injury as shown by a significant increase in ALT and bilirubin in severe cases of COVID‐19 (Guan et al., 2020). Therefore, it is likely that differentiating between COVID‐19‐induced transaminase elevations and those induced by remdesivir presents challenges (Bangash, Patel, & Parekh, 2020; Zhang, Shi, & Wang, 2020). However, a recent study found that only 4.1% of COVID‐19 patients receiving remdesivir treatment suffered serious (Grade 3 or 4) transaminase elevations, with there being no significant difference between the remdesivir‐ and placebo‐treated groups (Beigel et al., 2020). These data imply that remdesivir is relatively well tolerated in SARS‐CoV‐2‐positive patients. Regardless, as advised by the drug manufacturer, daily liver function tests are essential in any patients receiving remdesivir, with suggested discontinuation of the drug in patients whose ALT levels reach ≥5 times the upper limit of normal (ULN) (Gilead, 2020). Adhering to these guidelines is of particular importance in patients with pre‐existing liver disease or in those taking other medications that can also induce transient ALT and AST elevation (WHO, 2018).

The reported differences between preclinical and clinical data regarding the safety of remdesivir highlight the inadequacies of preclinical models in some contexts. For example, with regard to COVID‐19, a concerning element of theoretical toxicity is that which affects the respiratory system. A study using mouse models of Middle East respiratory syndrome coronavirus (MERS‐CoV) found that remdesivir improved pulmonary pathology in infected mice and rhesus monkeys, and no respiratory toxicity was observed (Gilead, 2020; Sheahan et al., 2020). In contrast, a respiratory safety study in rats showed that remdesivir had no effects on tidal volume or minute volume, but did increase respiratory rate, which returned to baseline by 24 h post‐dose (WHO, 2018). Clearly, increased respiratory rate is a manifestation of COVID‐19, and there would be problems in assessing causality if remdesivir was also likely to cause of respiratory problems in a clinical setting. Fortunately, a recent double‐blind, randomised, placebo‐controlled trial showed there to be no significant differences in adverse respiratory events between the remdesivir‐treated and control arms (Beigel et al., 2020). In addition to this, preclinical safety studies performed in rats and cynomolgus monkeys suggested that the kidney was the target organ for remdesivir‐induced toxicity (Gilead, 2020). This was a significant concern before the initial COVID‐19 clinical trials, as it is known that SARS‐CoV‐2 can cause acute kidney failure in severe cases (Ronco, Reis, & Husain‐Syed, 2020). However, this has not been reflected in COVID‐19 clinical trials, where the presence of biomarkers indicative of renal injury has not differed in patients treated with remdesivir, compared with those on placebo (Beigel et al., 2020; Gilead, 2020). However, due to the inclusion of the solubility enhancer sulfobutylether β‐cyclodextrin sodium (SBECD) within remdesivir formulations, remdesivir is contraindicated in patients with severe renal impairment (eGFR < 30 ml·min^−1^) (European Medicines Agency, 2020).

Finally, remdesivir is not exempt from DDIs. Co‐administration of remdesivir with several antibiotics including rifampicin is contraindicated, which could cause problems for any patients being treated concomitantly for tuberculosis (Liverpool Drug Interaction Group, 2020). This occurs because of enzyme induction that reduces systemic exposure to remdesivir. A similar interaction has also been seen with enzyme‐inducing anticonvulsants, including carbamazepine, phenytoin, and phenobarbital (Liverpool Drug Interaction Group, 2020), where reduction in remdesivir exposure may lead to inadequate treatment of COVID‐19.

### 3.4 Favipiravir

Favipiravir is another broad‐spectrum antiviral prodrug that undergoes intracellular phosphoribosylation to produce its active form, favipiravir‐ribofuranosyl‐5′‐triphosphate (favipiravir‐RTP) (Furuta, Komeno, & Nakamura, 2017). It is thought that this antiviral primarily acts by inducing lethal mutagenesis of RNA viruses, although it also selectively and potently inhibits viral RdRp by acting as a pseudopurine nucleotide (Dawes et al., 2018; Sangawa et al., 2013). Favipiravir is currently licenced in Japan for the treatment of novel and re‐emerging influenza (Furuta et al., 2013; Furuta et al., 2002). Its extensive spectrum of activity against various RNA virus polymerases led to favipiravir being cited as a potentially “crucial pandemic tool,” even before the outbreak of the novel coronavirus, COVID‐19 (Adalja & Inglesby, 2019).

The PK of favipiravir was initially characterised in healthy Japanese volunteers (Madelain et al., 2016). A *C*_max_ of 51.5 μg·ml^−1^ was found to occur 2 h post‐administration, but plasma concentrations decreased rapidly due to the relatively short half‐life of favipiravir (between 2 and 5.5 h) (Madelain et al., 2016). However, both *C*_max_ and half‐life increase slightly after multiple doses, and it has been suggested that favipiravir is capable of reaching a *C*_max_ in humans sufficient to inhibit 90% of SARS‐CoV‐2 replication, thus establishing it as an important compound in the ongoing search for COVID‐19 therapies (Arshad et al., 2020).

Marked differences in *C*_max_ have been observed between Japanese and American patients with *C*_max_ values in Japanese subjects being on average 13.26 μg·ml^−1^ greater than those in American subjects (Pharmaceuticals and Medical Devices Agency [PMDA], 2014). This highlights the need for relevant COVID‐19 clinical trials to include a diverse range of subjects so that factors such as weight and ethnicity can be considered to optimise dose. The bioavailability of favipiravir is high at 97.6%, and only 54% of the drug is plasma protein bound, suggesting that high tissue penetration would be likely (Madelain et al., 2016; PMDA, 2014). in vivo work in mice showed that the half‐life of favipiravir in the lungs is double than that of favipiravir in plasma, indicating slower elimination from the lungs (PMDA, 2014). This is thought to be of high importance in COVID‐19, where viral load is particularly high in the lungs. For influenza treatment in adults, 1,600‐mg favipiravir is given twice on Day 1 of treatment, followed by 600 mg twice daily from Days 2 to 5 (PMDA, 2014). However, the dosing period has been extended in ongoing COVID‐19 clinical trials: up to 10 days in ChiCTR2000029996 and 14 days in ChiCTR2000029548 (Guan et al., 2020). It is therefore essential that all PK parameters are monitored in these trials as differences, including increased *C*_max_ and decreased clearance, are expected during this prolonged dosing regimen that may affect safety.

Favipiravir has been linked to teratogenicity and embryotoxicity and is therefore contraindicated in pregnancy (Furuta et al., 2013). Overall, favipiravir is generally thought to have a good safety profile (Asrani, Devarbhavi, Eaton, & Kamath, 2019; Liverpool Drug Interaction Group, 2020; National Health Service [NHS], 2019). This is likely to be due to the fact that unlike other antiviral drugs such as ribavirin, favipiravir does not appear to disrupt nonviral RNA or DNA synthesis. However, very little is known about the long‐term safety of favipiravir, as in previous clinical trials, patient follow‐up has been as little as 5 days (Pilkington, Pepperrell, & Hill, 2020). This is perhaps less of a concern in COVID‐19 as treatment is time limited.

Drug–drug interactions have been reported with favipiravir. For example, coadministration with favipiravir can increase exposure to paracetamol by around 15%, which may be a concern for patients with pre‐existing liver disease, as paracetamol is the leading cause of acute drug‐induced liver injury (DILI) in the United Kingdom and United States (Asrani et al., 2019; Liverpool Drug Interaction Group, 2020). Favipiravir can also increase patient exposure to many contraceptives, including progesterone‐only pills, combined pills, and several contraceptive implants, which may cause discomfort, prolonged vaginal bleeding, and nausea (Liverpool Drug Interaction Group, 2020; NHS, 2019). Whether the increased exposure to oestrogens caused by concomitant treatment with favipiravir can enhance the risk of thrombosis is not known but should be monitored, given the overwhelming evidence that COVID‐19 increases the risk of blood clots (Atallah, Mallah, & AlMahmeed, 2020; Di Micco et al., 2020; Spiezia et al., 2020). Interestingly, large clots are most common in patients under the age of 50; almost 25% of women aged between 15 and 49 in the United States currently use either oral or long‐acting contraceptives and thus represent a particular risk group (Hurley, 2020; Centers for Disease Control and Prevention, 2019).

## 4 MODULATION OF THE INFLAMMATORY PHASE

SARS‐CoV‐2 virus is capable of eliciting an immune reaction in the infected individual. Laboratory tests have shown that inflammatory factors such as IL‐6, IL‐1, IL‐10, and TNF‐α are up‐regulated during infection and can instigate an inflammatory response in the lower airways leading to lung injury in some instances (Conti et al., 2020; Guo et al., 2020). Additionally, in patients with severe symptoms of COVID‐19, there may be activation of a cytokine storm, which can cause significant tissue damage (Mehta et al., 2020; Shi et al., 2020). A smaller proportion of patients can progress to a hyperinflammatory state, which in COVID‐19 has been suggested to resemble secondary haemophagocytic lymphohistiocytosis (sHLH), a rare syndrome characterised by uncontrollable fever, cytopenia, raised ferritin levels, and acute respiratory distress (Seguin, Galicier, Boutboul, Lemiale, & Azoulay, 2016). IL and TNF‐α levels show the greatest increase in those who require admission to the intensive care unit (ICU), suggesting that the cytokine storm is instrumental in severe COVID‐19 cases (Huang et al., 2020). Therefore, there has been a logical progression towards the use of immunosuppressive agents as potential therapies to alleviate inflammation and hyperinflammation associated with COVID‐19 (Mehta, McAuley, et al., 2020).

### 4.1 Glucocorticoids

Dexamethasone is a glucocorticoid that can be administered both orally and intravenously. It acts as a glucocorticoid receptor agonist and is over 20 times more potent than endogenous cortisol, thus resulting in dose‐dependent suppression of pro‐inflammatory genes through a number of pathways in common with other steroids (Papich, 2016; Whelan & Apfel, 2013; Yasir & Sonthalia, 2019). Low doses of glucocorticoids have an anti‐inflammatory effect, while higher doses are immunosuppressive (Buttgereit et al., 2002). Dexamethasone can be used for inflammatory diseases such as rheumatoid arthritis (Crohn's & Colitis Foundation, 2018; Freeman, 2008), but is recommended for short‐term treatment (spanning from 1 to 21 days) because of the major adverse effects that can occur with long‐term treatment. One of the commonest uses of dexamethasone is for reducing cerebral oedema.

As of July 8, 2020, dexamethasone was undergoing evaluation in 17 clinical trials. On June 16, the results of the dexamethasone arm of the RECOVERY trial were announced. The trial results, which are available in preprint form, showed that 2,104 patients had received either oral or intravenous low‐dose (6 mg) dexamethasone daily for 10 days (Horby et al., 2020). When compared with 4,321 control patients receiving usual care only, it was shown that dexamethasone reduced deaths by one third in SARS‐CoV‐2‐positive patients requiring ventilation and by one fifth in patients receiving oxygen. No benefit was observed for patients with milder COVID‐19 symptoms who did not require respiratory support (Horby et al., 2020).

Recent work has found that tissue inflammation and organ dysfunction seen in fatal cases of COVID‐19 are not consistent with SARS‐CoV‐2 distribution in tissues and cells (Dorward et al., 2020). Tissue‐specific tolerance to the virus may be therefore important and suggests that fatalities arising from COVID‐19 may be mainly due to host‐mediated immune response rather than pathogen‐mediated end‐organ inflammation. This is consistent with the dexamethasone result in the RECOVERY trial.

Dexamethasone has a bioavailability of 70–78% and is 77% protein bound in plasma (Spoorenberg et al., 2014). It is 6‐hydroxylated by hepatic CYP3A4 to 6α‐ and 6β‐hydroxy dexamethasone and can also be reversibly metabolised to 11‐dehydroxymethasone and back to dexamethasone by renal corticosteroid 11‐β‐dehydrogenase 1 (Diederich et al., 1998; Diederich, Hanke, Oelkers, & Bähr, 1997; Tomlinson, Maggs, Park, & Back, 1997). Unlike many glucocorticoids that are predominantly excreted in urine, only about 10% of dexamethasone is excreted in urine (Dexcel Pharma Technologies Ltd, 2019).

Glucocorticoids are generally safe drugs when given at low doses and for short periods of time (<3 weeks), with the risk of AEs increasing with dose and therapy duration (Yasir & Sonthalia, 2019). Short‐term use of dexamethasone can result in increased appetite, mood changes, and insomnia, but most of the adverse reactions are self‐limiting (NHS, 2020). Dexamethasone can lead to B‐ and T‐cell depletion and hence lymphopenia (Marinella, 2020), which interestingly is also found in up to 80% of patients with COVID‐19 (Liu, Blet, Smyth, & Li, 2020). However, despite this, the RECOVERY trial was able to show mortality benefit in the most severely affected COVID‐19 patients. A critical issue may be the dose that is administered—in RECOVERY, 6 mg·day^−1^ was administered over 10 days, which is a relatively low dose.

A recent systematic review and meta‐analysis of corticosteroid treatment in patients with coronavirus infection suggested that corticosteroids were associated with higher rates of bacterial infections, longer time spent in hospital, and higher rates of mortality (Yang, Liu, et al., 2020). However, most of the studies analysed in this meta‐analysis were retrospective observational studies, generally of poor quality and did not analyse the effects according to steroid dose. Other studies that have used low‐to‐moderate‐dose corticosteroids as treatment for diseases such as viral and bacterial pneumonia reflect the results of the RECOVERY trial, with low‐dose corticosteroids resulting in decreased mortality and morbidity in patients with severe pneumonia (Li et al., 2017; Stern et al., 2017). In these studies, low‐to‐moderate‐dose corticosteroids (40‐ to 50‐mg prednisolone, which equates to 6‐ to 7.5‐mg dexamethasone) were given to patients for between 7 and 10 days (Stern et al., 2017) (National Institute for Health and Care Excellence, 2020b). In keeping with the known adverse effects of corticosteroids, the systematic review showed that hyperglycaemia was significantly more frequent in the corticosteroid‐treated group (Stern et al., 2017).

Dexamethasone can be involved in both PK and pharmacodynamic interactions. Combining it with other immunosuppressants may increase the risk of serious infection (National Institute for Health and Care Excellence, 2020c). Co‐treatment with ibuprofen or other NSAIDs increases the risk of gastrointestinal bleeding (National Institute for Health and Care Excellence, 2020c), whilst its gluconeogenic effects can lead to hyperglycaemia, which in diabetic patients can lead to increased insulin doses being required (Consilient Health Ltd., 2020). Dexamethasone is a CYP3A4 inducer and may therefore interact with remdesivir, a CYP3A4 substrate, potentially reducing its plasma exposure. Although clinicians should be aware of this interaction, the risk is small given that both drugs are indicated for 10 days or less.

### 4.2 IL‐6 receptor inhibitors

Both tocilizumab and sarilumab are humanised anti‐IL‐6 receptor monoclonal antibodies used for the treatment of moderate to severe rheumatoid arthritis, whereas siltuximab is a chimeric, human–mouse anti‐IL‐6 receptor monoclonal antibody used for treatment of multicentric Castleman disease (MCD) (Deisseroth et al., 2015; National Institute for Health and Care Excellence, 2020e). Due to their long half‐life, IL‐6 inhibitors do not need to be taken daily; however, given that they are currently indicated for chronic diseases, patients receive IL‐6 inhibitor treatments for life or until treatment failure (Janssen Biotech Inc, 2019; Roche Pharma, 2013; Sanofi‐Aventis, 2017b).

Clinical trials to assess the efficacy and safety of tocilizumab, sarilumab, and siltuximab for the treatment of the inflammatory phase of COVID‐19 are ongoing. Whilst the exact dosing regimens vary between trials, COVID‐19 patients will be receiving a single or short‐course intravenous infusion or subcutaneous injection of the IL‐6 inhibitor (Clinical Trial Identifiers NCT04317092, NCT04315298, NCT04327388, NCT04330638, and NCT04322188).

Due to their similarity, it is not surprising that tocilizumab, sarilumab, and siltuximab have comparable safety profiles. Thus far, evidence from clinical trials in patients with rheumatoid arthritis and MCD or post‐marketing has revealed that IL‐6 inhibitors are generally well tolerated. Participants were enrolled on these trials for a minimum of 6 months and in some cases up to 24 months. Individuals with diabetes, a history of recurrent infection, age ≥65, and corticosteroid use have been shown to be at an increased risk of developing a more serious infection following IL‐6 inhibitor use (Jones et al., 2010). While adverse reactions were typically seen following chronic IL‐6 inhibitor treatment, the potential for COVID‐19 patients to develop an adverse drug reaction (ADR) following a single or small number of doses should not be ignored.

The most common infections reported in patients receiving anti‐IL6 therapy include skin infections, respiratory infections, urinary tract infections, and, in some cases, opportunistic infections ranging from tuberculosis to herpes (Emery et al., 2008; Emery et al., 2019; Fleischmann et al., 2017; Fleischmann et al., 2013; Genovese et al., 2015; Genovese et al., 2008; Genovese et al., 2019; Hoshi et al., 2012; Huizinga et al., 2014; Janssen Biotech Inc, 2019; Jones et al., 2010; Kameda et al., 2020; McCarty & Robinson, 2018; Pawar et al., 2019; Smolen et al., 2008; Tanaka et al., 2019; Weinblatt et al., 2013). Neutropenia has also been reported with all three drugs when tested for their intended therapeutic use, and in some trials, this led to patient discontinuation (Emery et al., 2008; Fleischmann et al., 2017; Fleischmann et al., 2013; Genovese et al., 2015; Genovese et al., 2008; Huizinga et al., 2014; Janssen Biotech Inc, 2019; Jones et al., 2010; Smolen et al., 2008). Absolute neutrophil count (ANC) must be monitored every 4–8 weeks, and in those who develop an ANC < 0.5 × 10^9^ L^‐1^, treatment must be discontinued (National Institute for Health and Care Excellence, 2020d; Roche Pharma, 2013; Sanofi‐Aventis, 2017b). Gastrointestinal manifestations (upper abdominal pain, mouth ulceration, and nausea) have also been reported with IL‐6 inhibitors. A systematic review and meta‐analysis of 35 COVID‐19 studies found that 15% of patients experienced gastrointestinal disturbance manifested as vomiting, diarrhoea, and loss of appetite. This has been postulated to be due to the expression of ACE2 on gastrointestinal epithelial cells (Mao et al., 2020; Qi, Qian, Zhang, & Zhang, 2020). Therefore, differentiating between COVID‐19‐induced gastrointestinal disorders and those evoked by IL‐6 therapy may present a challenge in terms of causality and the need to modify or stop therapy. Increases in lipid profiles (total cholesterol, LDL, HDL, and triglycerides) have also been reported in patients receiving IL‐6 inhibitors as either monotherapy or combination therapy (Emery et al., 2008; Fleischmann et al., 2013; Genovese et al., 2015; Genovese et al., 2008; Genovese et al., 2019; Huizinga et al., 2014; Janssen Biotech Inc, 2019; Jones et al., 2010; Kameda et al., 2020; Smolen et al., 2008; Tanaka et al., 2019). However, the incidence of major cardiovascular events was infrequent, and lipid elevation resolved with statin therapy (Genovese et al., 2015; Genovese et al., 2019).

Tocilizumab is associated with an increased risk of hepatotoxicity, usually manifested as an asymptomatic rise in ALT (Anger et al., 2017; Drepper, Rubbia‐Brandt, & Spahr, 2013; Fleischmann et al., 2013; Jones et al., 2010; Mahamid, Mader, & Safadi, 2011; Maini et al., 2006; Nishimoto et al., 2009; Pawar et al., 2019; Smolen et al., 2008). Liver injury has also been reported with a liver biopsy from a female patient who had taken tocilizumab for a month revealing focal necrosis of hepatocytes with steatosis and early fibrosis (Mahamid et al., 2011). COVID‐19 also has effects on the liver, and again, causality assessment may be difficult (Guan et al., 2020). The prescribing instructions for tocilizumab and sarilumab indicate that liver function tests are required every 4–8 weeks following treatment commencement and then every 3 months thereafter (Roche Pharma, 2013; Sanofi‐Aventis, 2017b). If liver enzymes are 1–3× ULN, the dose of tocilizumab and sarilumab can be reduced until ALT or AST have normalised and then treatment resumed at the therapeutic dose. Where laboratory findings are >3–5× ULN, treatment with IL‐6 inhibitors must be paused and then recommendations for 1–3× ULN followed. If elevations persist or are >5× ULN, tocilizumab and sarilumab treatment must be discontinued immediately (Roche Pharma, 2013; Sanofi‐Aventis, 2017b). Although sarilumab and siltuximab are associated with abnormalities in liver function tests, they are typically short lived and asymptomatic (LiverTox, 2016, 2017b). Pre‐existing liver disease can worsen symptoms of DILI and in some cases increase susceptibility (David & Hamilton, 2010).

Tocilizumab, sarilumab, and siltuximab are expected to undergo metabolism via catabolic pathways and not CYP450 processes (McCarty & Robinson, 2018). Therefore, due to the lack of hepatic metabolism, it is assumed that the PK of the IL‐6 inhibitors will not be altered in patients with pre‐existing liver disease (Abou‐Auda & Sakr, 2010). However, tocilizumab, sarilumab, and siltuximab have been shown to restore and improve CYP levels (Janssen Biotech Inc, 2019; Roche Pharma, 2013; Sanofi‐Aventis, 2017b). This is of particular importance as CYP levels may remain elevated following treatment discontinuation due to the long half‐life of the compounds. Therefore, this may be a consideration for further evaluation for any dosing adjustment requirements if patients are taking medications that are metabolised by CYP enzymes.

### 4.3 IL‐1 receptor inhibitors

Anakinra is a 17‐kD, recombinant human IL‐1 receptor antagonist that blocks the activity of pro‐inflammatory cytokines IL‐1α and IL‐1β (Cawthorne et al., 2011; Dinarello, Simon, & van der Meer, 2012). Anakinra is primarily used in combination with methotrexate for reducing the symptoms and slowing the progression of joint damage in rheumatoid arthritis (National Institute for Health and Care Excellence, 2020a). It is also used for rare inflammatory conditions such as cryopyrin‐associated periodic syndromes and Still's disease (National Institute for Health and Care Excellence, 2020a). It is administered via subcutaneous injection and is supplied as a single‐use, pre‐filled syringe containing 100 mg in 0.67 ml (Swedish Orphan Biovitrum Ltd, 2007). Rheumatoid arthritis patients and those with Still's disease and a body weight >50 kg must be given 100‐mg anakinra, whereas patients with Still's disease with a body weight <50 kg should have weight‐based dosing starting at 1–2 mg·kg^−1^ (Swedish Orphan Biovitrum Ltd, 2007). The recommended starting dose for patients with cryopyrin‐associated periodic syndromes is 1–2 mg·kg^−1^. If tolerated, the dose can be increased from 3–4 mg·kg^−1^ to a maximum of 8 mg·kg^−1^ (Swedish Orphan Biovitrum Ltd, 2007). Anakinra has a short terminal half‐life of approximately 4–6 h and so must be administered daily, preferably at the same time each day (Amgen Inc., 2001). Anakinra is currently not licenced for intravenous administration or treatment of sHLH, but its use is endorsed by clinicians, where intravenous infusion, as opposed to subcutaneous injection, can achieve quicker and greater maximal plasma concentrations (Carter, Tattersall, & Ramanan, 2018; La Rosée et al., 2019; Mehta, Cron, Hartwell, Manson, & Tattersall, 2020).

Thus far, 21 clinical trials have been registered to assess the use of anakinra in patients with severe COVID‐19. Additionally, two recent studies have reported positive outcomes with anakinra in COVID‐19‐induced acute respiratory distress syndrome (Cavalli et al., 2020; ClinicalTrials.gov, 2020c; Huet et al., 2020). Participants were dosed 100 mg twice daily subcutaneously for 72 h followed by 100 mg daily for 7 days in addition to standard of care (Huet et al., 2020). This retrospective study found that anakinra reduced rates of mortality and the need for mechanical ventilation in ICU patients (Huet et al., 2020). Anakinra was administered either subcutaneously or intravenously in the COVID‐19 Biobank Study (Huet et al., 2020). Participants received subcutaneous injections at a dose of 100 mg twice daily or via slow intravenous infusion at 10 mg·kg^−1^·day^−1^ until there was a 75% reduction in serum C‐reactive protein levels and sustained respiratory improvements (Cavalli et al., 2020). Alhtough no safety concerns emerged with anakinra administered subcutaneously, it was discontinued due to a lack of clinical improvement and limited reduction in C‐reactive protein (Cavalli et al., 2020). By contrast, intravenous anakinra was well tolerated and improved clinical outcomes. Notably, 72% of patients had improved respiratory function in comparison with 50% within the standard treatment group (Cavalli et al., 2020). In both studies, cases of ALT ≥ 3× ULN were observed in both the anakinra and the standard treatment arms. Four cases of bacteraemia following intravenous anakinra were reported in the COVID‐19 Biobank Study, but there were no cases of bacterial infection in the Ana‐COVID Study (Cavalli et al., 2020; Huet et al., 2020). While both studies are encouraging, they should be considered proof‐of‐concept trials, and larger randomised trials are still needed (Cavalli et al., 2020; Huet et al., 2020).

Subcutaneous administration of anakinra is associated with injection site reactions (Kaiser et al., 2012). In a review of five rheumatoid arthritis clinical trials, 71% of participants receiving anakinra therapy reported injection site reactions in comparison with 28% of participants on placebo (Mertens & Singh, 2009). Injection site reactions can range from immediate to delayed. In immediate cases, the reaction manifests as a burning sensation whereas delayed reactions present as a rash, pruritus, or swelling (Kaiser et al., 2012). Anakinra has also been reported to lead to infection, neutropenia, thrombocytopenia, headache, and blood cholesterol increase when administered subcutaneously (Swedish Orphan Biovitrum Ltd, 2007).

Injection site reactions that arise immediately can be eased by placing an ice pack on the injection site before and after anakinra administration, and delayed reactions can be treated with topical corticosteroids or antihistamines (Kaiser et al., 2012). Increases in serious infection rate are common following anakinra use and frequently include upper respiratory infections, sinusitis, urinary tract infection, and bronchitis (Bresnihan et al., 1998; Cohen et al., 2002; Fleischmann et al., 2003). Whilst rare, cases of opportunistic infection have been reported in anakinra monotherapy or in those receiving anakinra in combination with immunosuppressive agents (Salvana & Salata, 2009; Swedish Orphan Biovitrum Ltd, 2007). Neutrophil counts must be monitored during the first 6 months of anakinra treatment and quarterly henceforth (Swedish Orphan Biovitrum Ltd, 2007). In patients where the ANC is <1.5 × 10^9^ cells L^‐1^, treatment must be discontinued immediately (Swedish Orphan Biovitrum Ltd, 2007). The higher doses being used in COVID‐19 trials and the potential for a greater *C*_max_ due to intravenous administration potentially raise additional safety concerns. However, earlier detection of AEs should be possible since the duration of treatment will be shorter than that used in rheumatoid arthritis, coupled with the fact that patients will already be hospitalised.

Anakinra is catabolised and eliminated via glomerular filtration (Swedish Orphan Biovitrum Ltd, 2007; Yang, Baughman, & Sullivan, 2003). Caution should be exercised, and dose adjustments may be required in moderate to severe renal impairment (Swedish Orphan Biovitrum Ltd, 2007; Yang et al., 2003). During general infections and inflammatory diseases, CYP enzymes are primarily down‐regulated (Mallick, Taneja, Moorthy, & Ghose, 2017). Similar to IL‐6 inhibitors, it may be possible that anakinra treatment restores CYP levels in infected patients (Swedish Orphan Biovitrum Ltd, 2007). Therefore, caution should be exerted in COVID‐19 patients receiving concomitant medications with a drug having a narrow therapeutic window. Mild interactions can occur between anakinra and warfarin, clopidogrel, clozapine, and phenytoin (Liverpool Drug Interaction Group, 2020).

### 4.4 JAK inhibitors

Baricitinib is an orally administered, disease‐modifying, anti‐rheumatic drug (DMARD), traditionally used in the treatment of moderate to severe active rheumatoid arthritis (Al‐Salama & Scott, 2018). By acting as an ATP‐competitive kinase inhibitor, baricitinib can selectively and potently inhibit JAK1 and JAK2 in a reversible manner. JAKs are essential in the transduction of intracellular signals for various cytokines involved in the inflammatory and immune responses, and so by inhibiting these kinases, baricitinib is able to relieve symptoms of rheumatoid arthritis for many patients (Fridman et al., 2010).

As described previously, a common characteristic of COVID‐19, much like another betacoronavirus disease SARS, is a profuse inflammatory response (Huang et al., 2020; Stebbing et al., 2020). Increased levels of pro‐inflammatory cytokines, such as IFN‐γ and IL‐1β, have been observed in confirmed COVID‐19 cases (Huang et al., 2020; Mehta, McAuley, et al., 2020; Russell et al., 2020). Furthermore, the levels of some specific cytokines appear to be related to disease severity; patients requiring admission to intensive care units show increased levels of TNF‐α and the chemokine CCL2. The rationale behind repurposing baricitinib as a treatment for COVID‐19 is centred on this potential for severely ill patients to present with a cytokine storm (Mehta, McAuley, et al., 2020; Russell et al., 2020). By dampening the inflammatory response, it is postulated that baricitinib will be able to relieve COVID‐19 symptoms. Data modelled using artificial intelligence techniques suggest that baricitinib may work by inhibiting virus entry into cells via an endocytic regulator known to be involved in coronavirus internalisation, the AP2‐associated protein kinase 1 (AAK1) (Burkard et al., 2014; Richardson et al., 2020). Baricitinib, as well as being capable of JAK1 and JAK2 inhibition, is a high‐affinity inhibitor of AAK1 (Richardson et al., 2020).

Patients tend to tolerate baricitinib well, and it has a relatively good safety profile (Keystone et al., 2015). However, as with tocilizumab and sarilumab treatment, a very common (≥1/10) AE observed in patients taking baricitinib, but not in the placebo arm, is upper respiratory tract infection, which may be related to its ability to suppress the immune system (Eli Lilly, 2017). Patients taking baricitinib have the potential to develop respiratory tract infections, which may make it difficult to distinguish whether any deterioration is due to COVID‐19 or a secondary infection. Other opportunistic infections including herpes zoster and urinary tract infections were also more common in the treated arm compared with placebo, and dose reduction is recommended for patients with a history of chronic infections (Eli Lilly, 2017; Smolen et al., 2018). Secondary infections are not uncommon in severe COVID‐19 patients, and so the use of a drug that may make patients increasingly prone to infections will depend on the harm–benefit ratio for severe cases of COVID‐19 (WHO, 2020a).

Baricitinib is currently still being trialled in patients with COVID‐19 with a therapeutic dose of 2–4 mg once daily, which is the same as the recommended dosage for the treatment of rheumatoid arthritis (Cantini et al., 2020; Richardson et al., 2020). There have been a small number of reports from patients taking this recommended dosage for the treatment of rheumatoid arthritis presenting with deep vein thrombosis (DVT), which was severe in some of these cases (Taylor et al., 2019). This is a cause for concern as there are increasing reports of COVID‐19 patients, especially those who are critically ill and in the ICU, with thrombotic complications including pulmonary embolism and other venous and arterial thrombotic events (Klok et al., 2020; Middeldorp et al., 2020). As baricitinib has been reported to cause DVT, there is the potential for disease–drug interactions with COVID‐19 patients taking baricitinib potentially more likely to develop thrombotic complications. In order to mitigate this risk, alternative JAK inhibitors, which have a lower risk of thrombotic events, such as ruxolitinib, may be considered in the context of COVID‐19 (Alvarez‐Larran et al., 2018). However, unlike baricitinib, ruxolitinib is primarily metabolised by CYP3A4 (Yang & Keating, 2012). This means that prescribing ruxolitinib instead of baricitinib may increase the risk of CYP3A4‐related DDIs (Ogu & Maxa, 2000). Baricitinib is not predicted to be involved in any problematic DDIs. Co‐administration with both CYP3A inhibitors (fluconazole) and inducers (rifampicin) failed to result in any clinically relevant changes to baricitinib exposure (Eli Lilly, 2017).

Emerging reports have revealed that patients with COVID‐19 experience renal impairment, which could be attributed to ACE2 expression on kidney endothelial cells (Varga et al., 2020). Baricitinib should not be given to patients with renal impairment as the majority of the drug is cleared through the kidneys, and monitoring of renal function will be important to prevent AEs related to overexposure to baricitinib in those with deteriorating renal function (Eli Lilly, 2017).

## 5 STIMULATION OF THE BODY'S ANTIVIRAL RESPONSE

### 5.1 IFN‐β

Type 1 IFNs are a group of cytokines produced during viral infection. Notably, IFN‐β‐1a has a leading role in activating genes involved in immunomodulation, suppressing the inflammatory response and antiviral effects (Sallard, Lescure, Yazdanpanah, Mentre, & Peiffer‐Smadja, 2020). While a variety of type 1 IFNs exist, in vitro evidence has shown that IFN‐β‐1a and IFN‐β‐1b are the most potent in the inhibition of SARS‐CoV and MERS‐CoV (Chan et al., 2013; Hensley et al., 2004). Within the lungs, IFN‐β‐1 has been shown to up‐regulate levels of the enzyme CD73, an ecto‐5'‐nucleotidase, which inhibits vascular leakage, increases the secretion of anti‐inflammatory adenosine, and preserves pulmonary endothelial barrier function (Kiss et al., 2007; Sallard et al., 2020). However, in vivo research has revealed that timing of administration of IFN‐β‐1 is crucial for positive effects. When administered shortly after MERS‐CoV infection, IFN‐β‐1 protected mice from lethal infection, whereas delayed administration failed to effectively inhibit viral replication or pro‐inflammatory cytokines, leading to fatal pneumonia (Channappanavar et al., 2019). Interestingly, in vitro evidence has revealed that SARS‐CoV‐2 is more sensitive to IFN‐β‐1 treatment than MERS‐CoV and SARS‐CoV and thus supports the tenet that treatment with IFN‐β‐1 may be beneficial for COVID‐19 patients (Lokugamage, Schindewolf, & Menachery, 2020; Sheahan et al., 2020; Thiel & Weber, 2008). It is assumed that treatment of COVID‐19 patients with IFN‐β‐1 will strengthen the host immune response and prevent the worsening of severe respiratory tract manifestations.

IFN‐β‐1 therapy has been used for the long‐term management of multiple sclerosis (MS) and has been associated with a number of AEs. When administered subcutaneously in MS patients, the most common AEs were flu‐like symptoms, injection site reactions, worsening of MS symptoms, menstrual disorders, mood alterations, and laboratory abnormalities (Walther & Hohlfeld, 1999). The most common laboratory abnormalities were neutropenia, leukopenia, lymphopenia, and raised aminotransferases (Walther & Hohlfeld, 1999). A genome‐wide association study of patients with IFN‐β induced liver injury showed that rs2205986, which has been linked to differential expression of IFN regulatory factor (IRF)‐6, is a predisposing factor (Kowalec et al., 2018). This may be related to the fact that IRF6 leads to apoptosis in the presence of IFN‐β. Depression is a common AE reported in patients receiving subcutaneous IFN‐β‐1 therapy, and thus, caution is needed when administering to those with a previous or current history of depressive disorder (Biogen, 2007). Whilst rare, careful monitoring of clinical manifestations such as new onset hypertension, thrombocytopenia, impaired renal function, and fever is required in order to identify cases of thrombotic microangiopathy (TMA) (Biogen, 2007). TMA is rare and has been reported at different time points of IFN‐β‐1 therapy (Biogen, 2007; Nishio et al., 2016; Yam, Fok, McLean, Butler, & Kempster, 2018). Laboratory findings of a decreased platelet count, increased serum LDH, and red blood cell fragmentation are suggestive of TMA (Biogen, 2007). If diagnosed, patients must discontinue IFN‐β‐1 therapy and will require plasma exchange (Biogen, 2007).

SNG001 is an inhaled form of IFN‐β‐1a produced by Synairgen. The company has tested the efficacy and safety of the drug for the prevention and treatment of symptoms associated with respiratory viral infection in asthma and chronic obstructive pulmonary disease (COPD) (Synairgen plc, 2018). A randomised, placebo‐controlled, Phase 2 trial is currently ongoing to assess the safety and efficacy of inhaled SNG001 for the treatment of patients with COVID‐19 (NCT04385095). Data from the asthma trials have revealed that when administered via inhalation, high levels of IFN‐β‐1a are achieved within the lungs with lower levels within the circulation leading to improvements in lung function, antiviral responses, and better asthma control (Djukanović et al., 2014). Inhaled SNG001 seems to have a good safety profile; five patients within the SNG001 arm reported cardiac palpitations, whereas no cases were reported in the placebo arm, but symptoms were mild and not considered clinically significant (Djukanović et al., 2014).

A clinical trial has been undertaken in hospitalised COVID‐19 patients where the triple combination of IFN‐β, lopinavir–ritonavir, and ribavirin was compared with lopinavir–ritonavir and ribavirin (Hung et al., 2020; Shalhoub, 2020). Patients in the triple combination therapy arm achieved negative COVID‐19 tests results faster than those in the control arm, with improved patient symptoms, decreased viral shedding, and decreased overall length of stay in the hospital compared with those in the control group (Hung et al., 2020). AEs reported in both groups included nausea and diarrhoea. However, due to polypharmacy in this trial, it was difficult to determine the effect of IFN‐β on SARS‐CoV‐2 alone.

IFN‐β has reported DDIs with other COVID‐19 therapies including chloroquine and hydroxychloroquine and with anakinra, sarilumab, and tocilizumab (Liverpool Drug Interaction Group, 2020). DDIs have also been reported with metamizole (analgesic), linezolid (antibacterial), clozapine (antipsychotic), zidovudine (HIV antiretroviral therapy), and some immunosuppressants (adalimumab, azathioprine, and pirfenidone) (Liverpool Drug Interaction Group, 2020).

## 6 FUTURE OUTLOOK

Reviewing the safety of potential COVID‐19 treatments (Table 1) is complex due to the fast‐moving pace of research in this field. For example, chloroquine and hydroxychloroquine with or without an accompanying macrolide antibiotic have consistently been at the forefront of COVID‐19 research efforts since the outbreak began. However, the astonishing developments over a week or so have led to retraction of a highly publicised paper, and results from a post‐exposure prophylaxis trial and a treatment trial (RECOVERY), both of which have shown no beneficial effect of hydroxychloroquine (Boulware et al., 2020; Horby & Landray, 2020; Mehra, Ruschitzka, & Patel, 2020). This highlights that the rapid rate of discoveries surrounding COVID‐19 therapies generates the need to update this perspective frequently, in order to ensure that the safety of any newly repositioned therapies, novel developmental compounds or new therapeutic combinations are investigated. For example, the potential use of heparin in novel forms, including nebulised therapy (Clinical Trial Identifier NCT04397510), as an antiviral agent is currently the subject of several investigational trials. In addition, the potential utility of nitazoxanide is currently the subject of several clinical trials (ClinicalTrials.gov, 2020a; Pepperrell, Pilkington, Owen, Wang, & Hill, 2020; Rajoli et al., 2020).

**TABLE 1:** Overview of safety concerns of drugs considered for the treatment of COVID‐19

Drug class	Drug name	Mechanism	ADR—systemic symptoms, miscellaneous	ADR—organ system affected				
				Lung	Gastrointestinal	Liver	Kidney and urinary tract	Cardiovascular
Angiotensin receptor blockers	Losartan	Inhibitors of epithelium cell entry	Headache, dizziness, hypotension, fatigue, fetal toxicity	Yes?		ALT elevation, hepatotoxicity		Myocardial injury, atrial fibrillation
Anti‐HIV drug protease inhibitor	Lopinavir–ritonavir	Antiviral drug			Diarrhoea, nausea vomiting, pancreatitis			
Antimalarial	Chloroquine/hydroxychloroquine	Antiviral and immunomodulating effects	Retinal toxicity		Yes	Yes	Yes	QT prolongation, ventricular tachycardia
C‐adenosine nucleotide analogue developed for Ebola treatment	Remdesivir	Broad‐spectrum antiviral		Yes?		Mild‐to‐moderate ALT and AST elevation, LFT monitoring required	Kidney toxicity	
Pseudopurine nucleotide	Favipiravir	Broad‐spectrum antiviral	Teratogenicity, embryotoxicity	Bronchitis, cough		DILI with concomitant medications		Thrombosis with oestrogens
IL‐6 receptor inhibitors	Tocilizumab, sarilumab, siltuximab	Modulation of the hyperinflammatory phase, anti‐IL‐6 receptor monoclonal antibody	Neutropenia, increased infection rate, TB, herpes	Respiratory infection	Abdominal pain, mouth ulceration, nausea, lipid elevation, vomiting, diarrhoea, loss of appetite	Hepatotoxicity, LFT monitoring required	Urinary tract infection	
IL‐1 receptor inhibitors	Anakinra	Modulation of the hyperinflammatory phase, IL‐1 receptor antagonist	Infection, neutropenia, thrombocytopenia, headache, blood cholesterol increase, injection site reactions	Upper respiratory infection, bronchitis			Urinary tract infection	
JAK inhibitors	Baricitinib	Inhibition of virus entry into cells, anti‐inflammatory effect	Deep vein thrombosis				Exacerbation of renal impairment?	
Type 1 IFN	IFN‐β	Stimulation of the body's antiviral response	Neutropenia, leukopenia, lymphopenia, depression			Elevated ALT		
Glucocorticoid	Dexamethasone	Anti‐inflammatory	Increased appetite, mood change, insomnia, lymphopenia					

Abbreviations: ADR, adverse drug reaction; ALT, alanine aminotransferase; AST, aspartate transaminase; DILI, drug‐induced liver injury; HIV, human immunodeficiency virus; LFT, liver function test; TB, tuberculosis.

It is clearly essential that the harm–benefit ratio of any pharmacogical agent being considered for use in the treatment of COVID‐19 is thoroughly considered. This ratio changes dependent upon the disease stage and is correlated to potential mortality. For example, a higher risk may be acceptable for patients in the later stage of severe disease but not acceptable when the same therapeutic agent is administered in mild disease. This difference in harm–benefit analysis becomes even more striking when considering the use of such agents to prevent infection. As is the case for many highly contagious viruses, prevention by prophylaxis would be incredibly valuable. Some of the agents described in this review, including chloroquine and ritonavir, have been suggested as potential prophylactic agents, but to date, data on efficacy have been disappointing (Rathi, Ish, Kalantri, & Kalantri, 2020; Spinelli, Ceccarelli, Di Franco, & Conti, 2020). Clearly, treatment duration for prophylaxis is expected to be longer than for treatment of COVID‐19, and this may further alter the harm–benefit ratio, reinforcing the need for safety considerations at the outset of any clinical trials.

Similarly, the evaluation of therapy risk also applies to long‐term recovery. As the current pandemic progresses, it is becoming apparent that being discharged from hospital does not necessarily mean that patients are free from COVID‐19 symptoms. Large numbers of patients who have survived severe SARS‐CoV‐2 infection may have incurred long‐term health problems, including some permanent loss of lung and kidney function (Foundation, 2020; Su et al., 2020; Summers, 2020). Consequently, it is probable that long‐term therapies will be required for many patients to maintain or, ideally, restore normal physiological organ function. It is vital that therapies that will be used to treat patients during their long‐term recovery are also undergoing evaluation for their safety, particularly as many of these agents may need to be administered over much longer periods of time than initial COVID‐19 treatments.

The identification and characterisation of biomarkers of disease and safety will be invaluable in the further development and deployment of therapies for COVID‐19. Disease biomarkers, for example, of lung injury or the hyperinflammatory response, may allow the stratification of therapy in order to select the agent best suited to the stage of disease. Moreover, biomarkers should be considered to monitor patient safety in cases of known AEs. For example, the manufacturer's guidelines for remdesivir recommend daily liver function tests due to the risk of transaminase elevations (Gilead, 2020). These tests are essential, particularly with regard to COVID‐19 where increased ALT levels are reported to be common amongst hospitalised patients (Bangash et al., 2020; Zhang et al., 2009). Looking to the future, improvements in the specificity, predictivity, and reliability of drug‐induced organ damage, through academic–industry partnerships such as the Biomarker Qualification Program in the Critical Path Institute in the United States and the European Innovative Medicines Initiative consortium TransBioline, will help improve clinical assessment of drug safety issues in COVID‐19.

Continued enhancements in the speed, predictivity, and human translation of safety assessment for toxicity of antiviral compounds is clearly warranted, and this may include animal models of SARS‐CoV‐2 as well as in vitro models, in order to assess efficacy alongside safety. Such a full understanding for individual therapies will indicate the combinations that can have the potential to provide the best synergy for benefit, while forewarning of the potential for increased risk/harm through PK or toxicodynamic interaction.

Although outside the scope of this review, a vaccine for COVID‐19 remains the best hope to end the pandemic and protect the population. As of July 8, 2020, according to WHO, there are 21 vaccines in clinical trial stages and 139 in preclinical stages of evaluation (WHO, 2020b). Currently, potential vaccines are only just beginning to be tested for efficacy in humans in early phase studies, and therefore, safety data will begin to emerge as larger numbers of individuals are treated with the vaccine. Safety data regarding preliminary vaccinations against SARS and MERS are limited, but the available information may be useful during the development of COVID‐19 vaccines, due to the similarities between the coronavirus strains (Padron‐Regalado, 2020). One safety concern relevant to coronaviruses is the potential for the induction of antibody‐dependent enhancement (ADE), a phenomenon that was observed in cats vaccinated against feline infectious peritonitis coronavirus and has also been seen in patients vaccinated against Zika virus and dengue virus (Khandia et al., 2018; Padron‐Regalado, 2020; Vennema et al., 1990). ADE can occur when non‐neutralising antibodies bind to virus particles and increase their uptake into host cells, instead of rendering them non‐infectious (Padron‐Regalado, 2020; Tirado & Yoon, 2003). This caused concern in initial SARS vaccine development, but can reportedly be avoided by using truncated versions of the viral S glycoproteins (He et al., 2004). Acknowledging safety concerns such as this, as well as the ways they can be attenuated, may be paramount in the timely development of a vaccine against COVID‐19.

In conclusion, although expanding extremely rapidly, the field of therapies to treat COVID‐19 remains in its infancy. Safety will continue to play a major role in therapeutic success, as apparent with recent reports of increased cardiac toxicity associated with the use of chloroquine/hydroxychloroquine in the treatment of COVID‐19, despite its long history of use as an antimalarial. Above all, this perspective has exemplified the need to view safety concerns in the context of the individual and specific phase of disease in order to formulate a comprehensive harm–benefit balance. Importantly, an awareness of potential safety concerns will support the development of the next stage of therapy targeting prophylaxis and recovery post‐COVID infection. It is imperative that safety scientists rise to the challenge of COVID‐19 by utilising their expertise in mechanistic understanding, biomarker development, and toxicokinetic modelling in order to support the development of COVID‐19 therapies that can be used effectively and safely.

## NOMENCLATURE OF TARGETS AND LIGANDS

Key protein targets and ligands in this article are hyperlinked to corresponding entries in the IUPHAR/BPS Guide to PHARMACOLOGY (http://www.guidetopharmacology.org) and are permanently archived in the Concise Guide to PHARMACOLOGY 2019/20 (Alexander, Christopoulos, et al., 2019; Alexander, Cidlowski, et al., 2019; Alexander, Fabbro, et al., 2019a, b; Alexander, Kelly, et al., 2019; Alexander, Mathie, et al., 2019).

## CONFLICT OF INTEREST

A.A., B.K.P., C.B.‐B., C.E.G., R.L.J., R.T.K., S.H.K., S.L.P., and X.M. declare that they have no conflicts of interest. A.O. declares no direct conflict of interest but is Director and CSO for Tandem Nano Ltd and a co‐inventor of patents relating to drug delivery of infectious disease medicines. A.E.C. reports no direct conflict of interest but receives research funding for the support of S.L.P. and R.L.J. from Servier Pharmaceuticals and AstraZeneca; these are unrelated to the published work. A.E.C. receives additional unrelated research funding from Janssen Pharmaceuticals. A.O. has received consultancy and/or research funding from ViiV Healthcare, Merck, AstraZeneca, Gilead, and Janssen unrelated to the current paper. D.B. received educational grants and/or consultancy from AbbVie, Novartis, Merck, Gilead, and ViiV Healthcare outside the submitted work. M.P. receives research funding from various organisations including the MRC, NIHR, EU Commission, and Health Education England. He has also received partnership funding for the following: MRC Clinical Pharmacology Training Scheme (co‐funded by MRC and Roche, UCB, Eli Lilly, and Novartis) and a PhD studentship jointly funded by EPSRC and Astra Zeneca. He has also unrestricted educational grant support for the UK Pharmacogenetics and Stratified Medicine Network from Bristol‐Myers Squibb and UCB. None of the funding received is related to the current paper.
